# Evaluation of Sex Hormone Levels in Patients with Pemphigus Vulgaris in Comparison to the Healthy Population

**DOI:** 10.1155/2021/9947706

**Published:** 2021-09-28

**Authors:** Fatemeh Lavaee, Fahimeh Rezazadeh, Nasrin Saki, Zahra Tavazo, Saman Baghaei

**Affiliations:** ^1^Oral and Dental Disease Research Center, Oral and Maxillofacial Medicine, School of Dentistry, Shiraz University of Medical Sciences, Shiraz, Iran; ^2^Dermatology Department, Shiraz University of Medical Sciences, Shiraz, Iran; ^3^Student Research Committee, School of Dentistry, Shiraz University of Medical Sciences, Shiraz, Iran

## Abstract

**Materials and Methods:**

This cross-sectional study was performed on patients with pemphigus vulgaris referred to Faghihi Hospital and Shiraz Dental Faculty in 2017-2018. The participants included 26 women with histopathologically confirmed pemphigus vulgaris and 26 healthy age-matched controls. The serum levels of luteinizing hormone (LH), follicle-stimulating hormone (FSH), estrogen, progesterone, testosterone, prolactin, dehydroepiandrosterone (DHEA), and dihydrotestosterone (DHT) were evaluated in both groups. Independent *t*-test and two-way ANOVA were used for data analysis.

**Results:**

The mean age of the patients was 49.88 ± 10.46 years and that of the control group was 49.92 ± 11.30 years. Unlike the case group, the DHEA serum level was significantly higher among nonmenopausal participants in the control group. Moreover, the levels of testosterone and DHEA were significantly lower in the case group in comparison to the control group (*p* = 0.015 and *p* = 0.026, respectively).

**Conclusion:**

Considering the effects of age and menopause, the serum levels of testosterone and DHEA were significantly lower in the patients with pemphigus vulgaris than in the healthy controls. Hence, these hormones might have a role in the pathogenesis of pemphigus vulgaris.

## 1. Introduction

Pemphigus vulgaris is a common autoimmune blistering disease, i.e., the most common form of pemphigus, with the incidence of 2-10/1,000,000 [1, 2]. The incidence rate of pemphigus vulgaris in Iran has been reported as 5/100,000, being more common among females. The disease onset usually occurs during the fourth decade of life [3]. In this disease, autoantibodies are secreted against the intercellular adhesion proteins of desmosome. These autoantibodies are against desmoglein 1,3 in the mucocutaneous form and desmoglein 3 in the mucosal form of pemphigus vulgaris [4].

Generally, there is a clear sex tendency in the prevalence of most autoimmune diseases, with females being affected more [5]. Additionally, the onset of autoimmune diseases is associated with the levels of sex hormones. On the other hand, females go through several periods of hormonal change including puberty, menstruation period, pregnancy, and menopause. Thus, the prevalence of pemphigus vulgaris is higher among postmenopausal females. Estradiol and estrone levels decrease significantly during menopause. In fact, a marked decline in estradiol and estrone levels is the most prominent hormonal change in menopausal women. In addition to these changes, the serum levels of follicle-stimulating hormone (FSH) and luteinizing hormone (LH) increase and prolactin levels decrease slightly during menopause, while other pituitary hormones do not change. Besides, secretion of androgens decreases by aging. However, ovarian and adrenal glands secrete androgens both during and after menopause [6].

Hormonal changes in menopause cause general manifestations, which are concerning due to the increase in life expectancy. Decreased levels of progesterone, especially estrogen, and secondary alterations in FSH and LH are responsible for clinical manifestations postmenopause. In addition to the general manifestations of menopause such as hot flushes and psychological alterations, oral symptoms including burning mouth syndrome and dry mouth (xerostomia), some other disorders such as lichen planus, benign pemphigoid, and Sjogren's syndrome, and a rise in the prevalence of periodontal diseases have been reported in few studies [7]. There are also some immunological changes during menopause including increase in the production of and response to proinflammatory cytokines, decrease in the production of anti-inflammatory cytokines, and reduction in the activity of lymphocytes and natural killer cells. In addition to these changes, altered endocrine function may be responsible for increased incidence of autoimmune diseases with menopausal transition [5].

Up to now, several studies have been conducted on the relationship between sex hormones and induction of flares as well as treatment or even incidence of autoimmune diseases. For instance, some studies have indicated an association between menopause and age at the peak of rheumatoid arthritis incidence [8]. Besides, estrogen reduction could increase vascular diseases in systemic sclerosis [9]. Another study showed early menopause as a passive risk factor for the development of giant cell arteritis in postmenopausal women [10]. Moreover, some studies demonstrated the therapeutic effect of daily consumption of dehydroepiandrosterone (DHEA) on the improvement of the symptoms of Sjogren's syndrome and systemic lupus erythematosus (SLE) [11]. Nonetheless, to the best of the authors' knowledge, no study has evaluated the association between hormonal changes in menopause and higher prevalence of pemphigus vulgaris. Hence, the present study is aimed at evaluating this relationship among patients with pemphigus vulgaris.

## 2. Materials and Methods

### 2.1. Study Design and Patient Enrollment

This cross-sectional study was performed on patients with pemphigus vulgaris referred to Faghihi Hospital and Oral and Maxillofacial Disease Department of Shiraz Dental Faculty in 2017-2018.

The study protocol was concordant with the ethical principles of Helsinki (version 2002) and was approved by the Ethics Committee of Shiraz University of Medical Sciences (IR.SUMS.DENTAL.REC.1399.136).

Patients with histopathologically confirmed pemphigus vulgaris who were admitted or treated in the outpatient clinic were enrolled into the case group, and healthy age-matched women were considered as the control group. Since there was no similar study for sample size assessment, the results of the most similar study on an autoimmune disease was taken into account. Considering the power of 80%, type 1 error of 0.05, and correlation coefficient of 0.58, a 21-subject sample size was estimated for each group. Considering a 10% dropout, 26 participants were enrolled into each study group [11]. Prior to the beginning of the study, written informed consent forms were obtained from all participants.

The serum levels of sex hormones were assessed. In doing so, blood samples were obtained during routine evaluation before initiating corticosteroid therapy two hours after waking up on day three of the menstrual cycle. It is noteworthy that it was not necessary to specify a day for obtaining blood samples from menopausal women. The serum levels of LH, FSH, estrogen, progesterone, testosterone, prolactin, DHEA, and dihydrotestosterone (DHT) were evaluated via ELISA (monobind) Padtan Elm (EIA) kits (Saman Tajhiz, Iran) in Motahari laboratory. The participants' demographic data including age, disease extension, and menopause status were recorded, as well.

### 2.2. Statistical Analysis

The data were analyzed using the SPSS software, version 18. Independent *t*-test was utilized to compare the case and control groups regarding the mean levels of hormones. In addition, two-way ANOVA was used to compare the two study groups regarding the mean levels of hormones by taking the effect of age and menopause status into account. *p* < 0.05 was considered statistically significant.

## 3. Results

This study is aimed at evaluating the serum levels of sex hormones in 26 females with pemphigus vulgaris and 26 age-matched healthy controls. The mean age of the patients was 49.88 ± 10.46 years and that of the control group was 49.92 ± 11.30 years.

The mean serum levels of all the evaluated hormones have been presented in Table 1. As the table depicts, the levels of testosterone and DHEA were significantly lower in the case group in comparison to the control group (*p* = 0.015 and *p* = 0.026, respectively).

Considering the effect of age on the serum levels of sex hormones based on the results of two-way ANOVA, the difference between the two groups regarding sex hormones' serum levels have been presented in Table 2. Accordingly, the two groups were significantly different concerning the levels of testosterone and DHEA (*p* = 0.015 and *p* = 0.026, respectively).

The mean serum levels of sex hormones among menopausal and nonmenopausal participants in each study group have been shown in Table 3. The results revealed a significant difference between the menopausal and nonmenopausal patients with respect to the levels of FSH, LH, and estrogen (*p* = 0.001, *p* = 0.019, and *p* = 0.044, respectively).

Considering the effect of menopause status on the serum levels of sex hormones based on the results of two-way ANOVA, the difference between the menopausal and nonmenopausal participants was evaluated in each study group (Table 4). Accordingly, menopause had a significant effect on the levels of testosterone and DHEA (*p* = 0.017 and *p* = 0.047, respectively).

In comparison to the healthy controls, there were a larger number of patients with pemphigus vulgaris whose DHEA levels were within the normal range (*p* = 0.003) (Table 5).

## 4. Discussion

In this study, the serum levels of FSH and LH were significantly higher in menopausal participants than in nonmenopausal ones. However, the serum level of estrogen was significantly higher in nonmenopausal participants in both study groups. These differences were concordant with the effect of age on the natural changes in the serum levels of these hormones. In the control group, the DHEA serum level was significantly higher in nonmenopausal participants, while this was not the case among the patients with pemphigus vulgaris, which might indicate some hormonal disturbances in these patients. Considering the effect of age on the serum levels of sex hormones, significantly lower levels of DHEA and testosterone were observed in the patients with pemphigus vulgaris compared to the healthy controls. In this comparison, although the *p* value for estrogen was not statistically significant, it was considerable in both the effect of menopause (*p* = 0.061) and the effect of age (*p* = 0.054) on the serum levels of sex hormones. In case a larger number of participants were enrolled into the case group, significant results might have been obtained.

The previous studies assessed the role of sex hormones in immune system regulation, activation, or suppression. Accordingly, there are several theories on the effects of these hormones on autoimmune situations. However, these theories are sometimes controversial. Considering pemphigus vulgaris as an autoimmune disease, researchers have made attempts to evaluate the role of sexual hormones in the pathogenesis of this disease. To the best of the authors' knowledge, no similar study has been conducted on pemphigus vulgaris. Nonetheless, other autoimmune diseases have been evaluated in both human and animal models using different methods and concepts. For instance, Forsblad-d'Elia et al. evaluated the effect of daily consumption of 50 mg oral DHEA in primary Sjogren among postmenopausal women. They reported the relief of the dry mouth symptom as well as an increase in the levels of testosteron, estrogen, and DHEA in the case group in comparison to the placebo group [11]. These results were in agreement with those of the current investigation although the methodologies were different.

The current study findings revealed that the serum levels of DHEA (*p* = 0.026) and testostrone (*p* = 0.015) were significantly lower in the patients affected by pemphigus than in the healthy controls. This was consistent with the results of similar studies on Sjogren's syndrome [11–13], thyroid autoimmune diseases [14], rheumatoid arthritis [15, 16], and systemic lupus erythematosus [17], which showed androgen deficiency (DHT, DHEA-S, etc.) in these participants [12].

There are some reports about the possible interrelation between DHEA and soluble immune mediators, which can affect the function and adhesion of leukocytes in females with SLE [17]. In another study, the potential neuroprotective effects of testosterone therapy were explored in males with relapsing-remitting multiple sclerosis [18]. The suppressive activity of the immune system after a single-dose injection of testosterone was also reported in an animal model [19]. Yet, there are some controversies regarding the effects of testosterone and DHEA (androgen) in patients with SLE. Although testosterone improved SLE complications in a specific period of evaluation in the research performed by Van Vollenhoven et al., this finding was not repeated in other phases of the study [20–22]. In addition, another research showed no significant improvement in the severity of SLE [23].

Studies on sex hormones are not limited to androgens and testosterone. A prior study indicated no significant relationships between exposure to estrogen or prolactin and increased risk of lupus. That study as well as other studies assumed that early menopause could be associated with an increased risk of SLE development [24, 25]. Moreover, the anti-inflammatory property of estrogen has been found to have protective effects on inflammatory bowel disease [26].

A previous study revealed an immunosuppressant activity, especially delayed type of hypersensitivity, for estriol. This hormone therapy was suggested for treating multiple sclerosis [27]. Although reports on the positive effects of estradiol are more prominent, the reverse can be found in some investigations [28, 29]. On the other hand, Hazes et al. disclosed no significant improvement in the symptoms of rheumatoid arthritis after estrogen therapy [29]. Yet, another research suggested antiprolactin and estrogen medications for relieving SLE complications [28].

The adrenal glands in humans secrete DHEA, especially DHEA-S, which can transform to androgen or estrogen in peripheral tissues [30]. Thus, these hormones play an important role in menopausal women with no ovarian estrogen secretion. It should be noted that adrenal glands are the only active source of DHEA secretion in females, while testes secrete androgen in males throughout life [30]. In the present study, DHEA-S was reduced in the healthy postmenopausal participants as well as in the patients with pemphigus vulgaris. However, this reduction was more prominent in the case group. The more significant decrease in the serum level of DHEA could be attributed to the greater risk of pemphigus vulgaris development in females, especially during the postmenopause period. Medications and adrenal diseases inducing adrenal suppression should also be considered along with this possible association. Overall, DHEA administration in postmenopausal women has been considered beneficial for the bone, skin, and well-being for many years [31, 32].

Testosterone can be secreted by the ovaries, adrenal glands, and peripheral tissues. The proportion of testosterone secretion from these sources can vary by aging. In fact, the serum level of this hormone is affected by aging rather than by menopause. Thus, a woman in her forth decade of life has half of the testosterone level as a woman in her second decade of life [33, 34]. The current study findings showed no significant difference between the menopausal and nonmenopausal participants in the case and control groups regarding the serum level of testosterone. However, a more prominent decreasing trend was observed in this hormone among the patients with pemphigus vulgaris. It is worth mentioning that the testosterone level can be affected by adrenal suppression and medications.

Menopause exerts a negative impact on women's health. This situation that is associated with a progressive decrease in sex steroids followed by estrogen secretion arrest can be related to many morbidities. In fact, a marked decline in estradiol and DHEA after menopause is a critical challenge among females. The present study results indicated no significant reduction in the estrogen serum level among the patients with pemphigus vulgaris. Yet, choosing a larger sample size might have led to more precise results.

The present study had some limitations related to the small sample size and patient selection. Considering a healthy control group, using a larger sample size, evaluation of all effective factors in hormone levels could lead to more reliable results. Furthermore, a controlled study is recommended to be performed on the effect of hormone therapy using estrogen in an animal in order to explore the risk of endometrial cancer. Hormone therapy or balancing sex hormones, especially DHEA and testosterone, that were more significant in this study can provide a new insight for controlling, preventing, or even treating pemphigus vulgaris, which should be evaluated precisely in future studies.

## 5. Conclusions

Considering the effects of age and menopause status, the serum levels of testosterone and DHEA were significantly lower in the patients with pemphigus vulgaris than in the healthy participants. Therefore, these hormones may play a role in the pathogenesis of pemphigus vulgaris.

## Figures and Tables

**Table 1:** The mean serum levels of all sex hormones.

Sex hormone	Patients (mean)	Controls (mean)	*p* value
Age (years)	49.8846	49.9231	
FSH (mlu/ml)	46.4246	35.9512	0.115
LH (mlu/ml)	19.2885	14.7723	0.162
PRL (ng/ml)	20.8769	15.6154	0.231
TESTO (ng/ml)	0.2477	0.4058	0.015
DHEA (*μ*g/ml)	54.0769	91.2692	0.026
EST (pg/ml)	54.2192	73.5885	0.059
PROG (ng/ml)	0.4631	1.1115	0.117
DHT (pg/ml)	214.1154	221.0385	0.802

PRL: prolactin; TESTO: testosterone; DHEA: dehydroepiandrosterone; EST: esteradoil; PRO: progesterone; DHT: dihydrotestosterone.

**Table 2:** The difference between the two groups regarding the serum levels of sex hormones considering the effect of age.

Hormone	FSH	LH	PRL	TESTO	DHEA	EST	PRO	DHT
Case vs. control serum level (*p* value)	0.115	0.162	0.231	0.015	0.026	0.059	0.117	0.802

PRL: prolactin; TESTO: testosterone; DHEAS: dehydroepiandrosterone; EST: esteradoil; PRO: progesterone; DHT: dihydrotestosterone.

**Table 3:** The mean serum levels of sex hormones in the menopausal and nonmenopausal participants in each study group.

Group	*N*	Mean	Std. deviation	Std. error of mean	*p* value
Patients					
FSH (mlu/ml) nonmenopause Menopause	13 13	27.0254 65.8238	30.97540 17.48541	8.59103 4.84958	0.001
LH (mlu/ml) nonmenopause Menopause	13 13	12.9608 25.6162	15.16710 9.95006	4.20660 2.75965	0.019
PRL (ng/ml) nonmenopause Menopause	13 13	28.0000 13.7538	24.84720 5.23635	6.89137 1.45230	0.054
TESTO (ng/ml) nonmenopause Menopause	13 13	0.2031 0.2923	0.17231 0.22924	0.04779 0.06358	0.273
DHEA (*μ*g/ml) nonmenopause Menopause	13 13	57.6923 50.4615	82.01360 50.58098	22.74648 14.02864	0.789
EST (pg/ml) nonmenopause Menopause	13 13	63.1769 45.2615	26.14381 15.55986	7.25099 4.31553	0.044
PROG (ng/ml) nonmenopause Menopause	13 13	0.4615 0.4646	0.27300 0.21812	0.07572 0.06050	0.975
DHT(pg/ml) nonmenopause Menopause	13 13	174.7692 253.4615	38.65694 151.31678	10.72151 41.96772	0.091
Controls					
FSH (mlu/ml) nonmenopause Menopause	13 13	9.8746 62.0277	14.46606 25.96901	4.01216 7.20251	<0.001
LH (mlu/ml) nonmenopause Menopause	13 13	7.2146 22.3300	10.72106 9.50744	2.97349 2.63689	0.001
PRL(ng/ml) nonmenopause Menopause	13 13	12.6615 18.5692	6.75691 13.94300	1.87403 3.86709	0.182
TESTO (ng/ml) nonmenopause Menopause	13 13	0.4777 0.3338	0.27782 0.20851	0.07705 0.05783	0.148
DHEA (*μ*g/ml) nonmenopause Menopause	13 13	127.4615 55.0769	79.34483 32.10000	22.00630 8.90294	0.008
EST (pg/ml) nonmenopause Menopause	13 13	103.8846 43.2923	61.54456 13.91633	17.06939 3.85969	0.004
PROG(ng/ml) nonmenopause Menopause	13 13	1.8308 0.3923	2.83661 0.25824	0.78673 0.07162	0.093
DHT(pg/ml) nonmenopause Menopause	13 13	240.3077 201.7692	74.82355 77.16341	20.75232 21.40128	0.208

PRL: prolactin; TESTO: testosterone; DHEA: dehydroepiandrosterone; EST: esteradoil; PRO: progesterone; DHT: dihydrotestosterone.

**Table 4:** The difference in the serum levels of sex hormones considering the effect of menopause.

Hormone	FSH	LH	PRL	TESTO	DHEA	EST	PROG	DHT
Case vs. control serum level (*p* value)	0.110	0.162	0.230	0.017	0.047	0.061	0.118	0.801

**Table 5:** The number of participants with normal and abnormal levels of sex hormones in the case and control groups.

	Normal range		Abnormal range		*p* value
	Case	Control	Case	Control	
FSH (mlu/ml)	21	23	5	3	0.7007
LH (mlu/ml)	22	24	4	2	0.6642
PRL (ng/ml)	13	10	13	16	0.5766
TESTO (ng/ml)	0	0	26	26	0.0001
DHEAS (*μ*g/ml)	12	23	14	3	0.003114
EST (pg/ml)	21	22	5	4	0.999
PROG (ng/ml)	26	24	0	2	0.4708
DHT (pg/ml)	5	2	21	24	0.4164

PRL: prolactin; TESTO: testosterone; DHEAS: dehydroepiandrosterone; EST: esteradoil; PRO: progesterone; DHT: dihydrotestosterone.

## Data Availability

The readers can access the data supporting the conclusions of the study by a request through an email to the corresponding author.
